# The cardiovascular effects of air pollution: Prevention and reversal by pharmacological agents

**DOI:** 10.1016/j.pharmthera.2021.107996

**Published:** 2022-04

**Authors:** Mark R. Miller

**Affiliations:** University/BHF Centre for Cardiovascular Sciences, University of Edinburgh, Edinburgh, Scotland, UK

**Keywords:** Air pollution, Particulate matter, Diesel exhaust, Cardiovascular, Antioxidant, ACE, angiotensin converting enzyme, ApoE, apolipoprotein-E, CAPs, concentrated ambient particles, DEP, diesel exhaust particulate, ECG, electrocardiogram, eNOS, endothelial nitric oxide synthase, ET-1, endothelin-1, GSTM1, glutathione-S-transferase Mu 1, HRV, heart rate variability, ICAM-1, intercellular adhesion molecule-1, iNOS, inducible nitric oxide synthase, LDL, low density lipoprotein, NAC, *N*-acetylcysteine, NAD(*P*)H, nicotinamide adenine dinucleotide phosphate, NO, nitric oxide, NO_2_, nitrogen dioxide, NOS, nitric oxide synthase, oxLDL, oxidised low density lipoprotein, PM, particulate matter, PM_0.1_, particulate matter with a diameter of less than 0.1 μm (nanoparticles), PM_10_, particulate matter with a diameter of 10 μm or less, PM_2.5_, particulate matter with a diameter of 2.5 μm or less, sGC, soluble guanylate cyclase, SOD, superoxide dismutase, TNF_α_, tumour necrosis factor alpha, TRPV1, transient receptor potential cation channel V1, t-PA, tissue plasminogen activator, VCAM-1, vascular cell adhesion molecule-1, vWF, von Willebrand Factor

## Abstract

Air pollution is associated with staggering levels of cardiovascular morbidity and mortality. Airborne particulate matter (PM), in particular, has been associated with a wide range of detrimental cardiovascular effects, including impaired vascular function, raised blood pressure, alterations in cardiac rhythm, blood clotting disorders, coronary artery disease, and stroke. Considerable headway has been made in elucidating the biological processes underlying these associations, revealing a labyrinth of multiple interacting mechanistic pathways. Several studies have used pharmacological agents to prevent or reverse the cardiovascular effects of PM; an approach that not only has the advantages of elucidating mechanisms, but also potentially revealing therapeutic agents that could benefit individuals that are especially susceptible to the effects of air pollution. This review gathers investigations with pharmacological agents, offering insight into the biology of how PM, and other air pollutants, may cause cardiovascular morbidity.

## 1 Introduction

History has demonstrated the deadly consequences of air pollution: the 1930 Meuse Valley fog in Belgium saw a 3-day persistence of industrial emissions that resulted in 60 deaths; 3 days of high air pollution from the 1948 Donora air inversion in the USA led to ill-health in over one-third of the town's population; the 5-day period of high air pollution of the 1952 London smog in the United Kingdom is estimated to have caused between 4000 and 10,000 deaths. While these events have not been forgotten, the awareness of the health effects of air pollution has grown considerably over the last decade. Today, governments in both developed and developing countries alike have declared their commitment to tackle the issue. Yet despite this attention, the health effects of air pollution persist at staggering levels, with estimates that air pollution is responsible several million premature deaths globally every single year (Lim et al., 2012; World Health Organization, 2014). Indeed, using 2015 data, the Global Burden of Disease group ranked ambient (outdoor) air pollution as the fifth biggest risk factor for all-cause disease (Cohen et al., 2017). It is clear now that air pollution has effects far beyond the lung; in almost all areas of the body, in fact (Schraufnagel et al., 2019a, Schraufnagel et al., 2019b). However, the cardiovascular effects of air pollution are especially prominent, with ischaemic heart disease and stroke accounting for approximately a half of the early deaths attributed to air pollution (Burnett et al., 2018; Cohen et al., 2017; Lelieveld et al., 2019).

The biological mechanisms underlying the link between air pollution and cardiovascular disease has been the subject of intense research over the last two decades. It is now recognised that the small particles in air pollution can have detrimental effects throughout the cardiovascular system; on the heart, the vasculature and the blood (Miller & Newby, 2020). Substantial progress has also been made in identifying the biological pathways by which particles inhaled into the lung then progress to effects on the cardiovascular system (Miller, 2014, Miller, 2020; Miller & Newby, 2020; Munzel et al., 2017). This mechanistic knowledge has practical value as it sheds light on which pollutants are likely to be the most harmful, who may be the most susceptible, and reveals the parameters with which to measure to investigate the potential benefits of interventions. Yet the sheer volume of data amassed has also brought the new challenge of trying to ascertain which mechanisms are the key drivers for pathophysiological effects. The complexity of the underlying biological processes involved in the health effects of air pollution, and their many interactions, has left researchers with a vast maze of pathways to negotiate. To this end, this review gathers together research that has used pharmacological agents to prevent or reverse the cardiovascular effects of air pollutants, especially those caused by inhaled particles. While reducing air pollution should undoubtedly be the primary route to reduce its health impact, a safe cost-effective pharmacological intervention could have value in ameliorating the effects of air pollution in those that are especially susceptible to the actions of pollutants and/or have an unavoidable high exposure. Additionally, one of the key advantages of pharmacological studies is that they offer an additional degree of certainty on the issue of causality of specific biological pathways underlying any observed associations. Thus, pharmacological approaches may provide some useful signposts through the biological labyrinth of air pollution and cardiovascular disease.

## 2 What is air pollution?

**Fig. 1:**
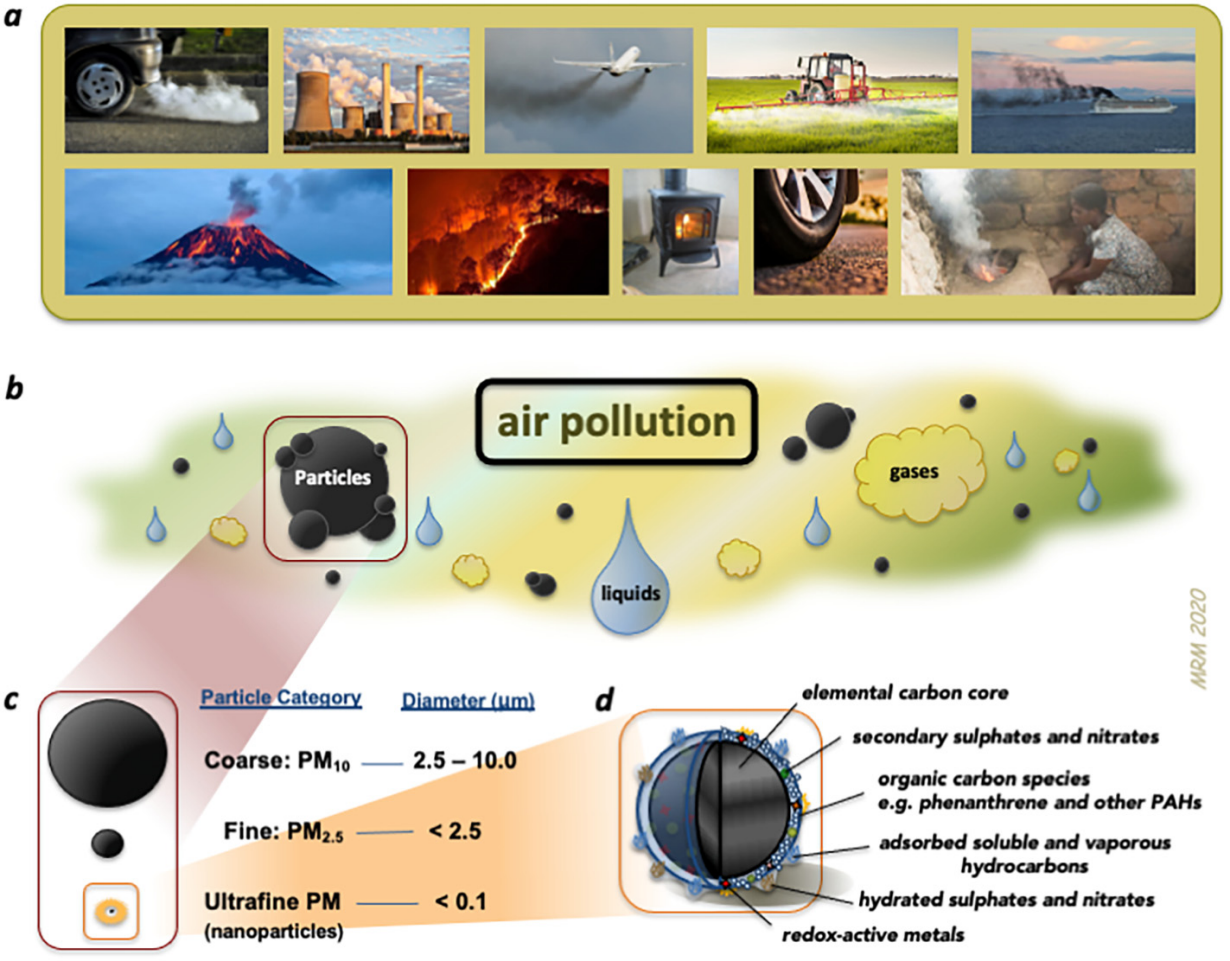
Composition and categorization and air pollution and particulate matter. *a*. Examples of key sources of air pollution, which can be both natural or anthropological. *b*. Air pollution can be broadly characterized into gases, volatile liquids and particles. *c*. Different size categories of PM. *d*. Schematic showing the complex composition of a combustion-derived nanoparticle such as diesel exhaust particulate. Adapted from (Miller & Newby, 2020).

There are many different sources of air pollution in our environment; from natural sources such as dust storms, wild-fires and volcano eruptions, to anthropological sources such as agriculture, crop-burning, industry, heating, cooking and vehicle emissions. While biomass burning is a prominent source of air pollution in developing countries, the focus of the majority of global research attention has been on urban pollutants and those from combustion of fossil fuels. Urban air pollution is a complex mixture of many different gases, volatile liquids and particulates (Niemann et al., 2017) (Miller, 2020). People are exposed to a mixture of air pollutants, varying over time and between environments, and the complexities of this mixture and the biological responses present a challenge in identifying which specific pollutants may be most harmful to health. Both gaseous pollutants (such as ozone, nitrogen oxides and sulphur dioxide) and particulates have detrimental effects on cardiovascular health, however, epidemiology studies have found the greatest and most robust associations for the particulate matter (PM) in the air (Brook et al., 2010). Urban PM is a highly heterogenous mixture of particulates arising from many sources and containing particles of different sizes and composition. These characteristics of particles influence their potential toxicity, for example, by determining the ability of the particle to access different regions of the body (Raftis & Miller, 2019), the chemical reactivity of the particle and the consequent actions of the particle in the biological compartment (Miller, 2020) (see Section 4). PM in the environment is monitored by using a mass metric of particles of certain sizes, with PM_10_ and PM_2.5_ representing particulate matter with a diameter smaller than 10 or 2.5 μm, respectively. A third category of particles is ultrafine particles (PM with a diameter of 100 nm of less: PM_0.1_ or ‘nanoparticles’) which cannot currently be measured routinely outside the laboratory on any great scale. Th The very small size of ultrafine PM means it has a low mass and is not adequately measured by PM_10_ or PM_2.5_ metrics. However, the small particle size and high surface area engenders the particles with a high capacity to cause detrimental health effects (Ohlwein, Kappeler, Kutlar Joss, Kunzli, & Hoffmann, 2019). In urban environments, a significant proportion of PM_2.5_ and ultrafine PM is derived from combustion of fuels, and vehicle exhaust in particular is a major source of ultrafine PM (Geller et al., 2006; Steiner, Bisig, Petri-Fink, & Rothen-Rutishauser, 2016). The complex composition of urban PM also plays a major role in toxicity. Combustion-derived nanoparticles, such as diesel exhaust particulate (DEP), are primarily composed of an elemental and organic carbon core, with a vast mixture of many thousands of different chemicals adsorbed to its surface (Alves et al., 2015; Schuetzle et al., 1994; Steiner et al., 2016; Wichmann, 2007) (Fig. 1). Organic carbon species and reactive transition metals on the surface of DEP are believed to be key drivers of their toxicity (Cassee, Heroux, Gerlofs-Nijland, & Kelly, 2013; Gerlofs-Nijland et al., 2009; Miller, Shaw, & Langrish, 2012; Schwarze et al., 2007; Steiner et al., 2016).

## 3 Air pollution and cardiovascular disease

While the respiratory effects of air pollution have been recognised for many decades, the last two decades of research have cemented cardiovascular disease as a significant consequence of air pollution. Both acute and chronic exposure to air pollution has been implicated in a wide range of cardiovascular conditions, including myocardial infarction, heart failure, hypertension and stroke (Brook et al., 2010; Miller & Newby, 2020). Epidemiological studies have more consistently shown relationships between these conditions with particulate components, as compared to gaseous constituents, although there is still a substantial body of evidence showing detrimental effects of gases such as nitrogen dioxide (NO_2_) in cardiovascular conditions (Brook et al., 2010; Newby et al., 2015). Long-term exposure to PM_2.5_ has been shown to be associated with both all-cause mortality and cardiovascular mortality (Hoek et al., 2013; Lelieveld et al., 2020; Pope III et al., 2004; Pope III et al., 2019; Pun, Kazemiparkouhi, Manjourides, & Suh, 2017; Wang et al., 2020). Furthermore, endpoints such as carotid intima thickness and coronary artery calcification, have linked air pollution exposure with chronic conditions like atherosclerosis (Hoffmann et al., 2007; Kunzli et al., 2005; Provost, Madhloum, Int Panis, De Boever, & Nawrot, 2015). Associations tend to be more robust for PM_2.5_ than PM_10_ (Brook et al., 2010; Liu et al., 2019), suggesting PM_2.5_ is also responsible for a substantial proportion of the effects of PM_10_. Due to the difficulty of measuring ultrafine particles at a population level, epidemiological evidence is limited for this PM category, although using particle number as a surrogate for ultrafine PM supports associations with cardiovascular disease (Stone et al., 2017).

Controlled exposure studies provide a useful means to explore the actions of specific pollutants without many of the confounders of epidemiological studies. In these investigations, a volunteer inhales a specific pollutant at a controlled level for a short period of time (usually 1–3 h) within a chamber or directly to the nose and mouth. Exposures can be concentrated ambient particles (CAPs) or purer sources of pollutants such as diluted vehicle exhaust. Our own group carried out a programme of studies in both healthy volunteers and patients with cardiovascular disease, with participants being exposed to dilute diesel exhaust to study the cardiovascular effects of combustion-derived nanoparticles (Mills et al., 2009). Acute exposure (1 or 2 h) to diesel exhaust at a concentration representative of traffic-congested city centre roads had profound actions across the cardiovascular system. Exposure impaired vascular relaxation responses (Mills et al., 2005), increased arterial stiffness (Lundback et al., 2009), promoted blood clotting (Lucking et al., 2008), impaired fibrinolysis (Mills et al., 2005) and increased ischaemic stress in the heart (Mills et al., 2007). Other studies also found that controlled exposure to diesel exhaust impaired microvascular function (Wauters et al., 2013) and increased blood pressure (Cosselman et al., 2012; Tong et al., 2014). There are also isolated studies demonstrating that diesel exhaust may alter the rhythm of the heart (Tong et al., 2014), although such effects are more common to other forms of urban PM than pure vehicle exhaust in human studies (Mills et al., 2011). The particulate elements of diesel exhaust drives these acute cardiovascular effects, as exposure of gaseous pollutants in isolation (e.g. NO_2_ or ozone (O_3_)) do not induce cardiovascular changes (Barath et al., 2013; Langrish et al., 2010), and removal of particulates from the whole exhaust prevents the acute cardiovascular impairment (Lucking et al., 2011; Mills et al., 2011). Additionally, animal studies have shown that particulate components of urban PM or vehicle exhaust have the capacity to promote growth of atherosclerotic plaques, as well as increase markers of vulnerability to plaque rupture (plaque rupture may be the trigger for a heart attack, stroke or embolism) (Bai et al., 2011; Campen et al., 2010; Miller et al., 2012; Miller et al., 2013; Moller et al., 2011). Overall, PM, and combustion-derived nanoparticles in particular, have the capacity to cause multiple types of dysfunction throughout the cardiovascular system, with the potential to instigate early events in disease, exacerbate existing disease processes and potentially even trigger the acute cardiovascular events associated with mortality.

## 4 Biological pathways

The cellular pathways mediating the cardiovascular effects of PM are many and varied (see Miller & Newby, 2020; Munzel et al., 2017 for more detailed description). Oxidative stress and inflammation are hallmarks of pollutant exposure both in the lung and throughout the cardiovascular system (Kelly & Fussell, 2017; Miller, 2020; Miller et al., 2012). Urban PM and DEP have the capacity to generate oxygen free radicals from the particle surface (Briede et al., 2005; Ikeda, Suzuki, Watarai, Sagai, & Tomita, 1995; Miller et al., 2009). Furthermore, a number of components within PM can disrupt cellular homeostasis, inducing the formation of free radicals from the mitochondria and a series of cellular enzymes, such as nicotinamide adenine dinucleotide phosphate (NAD(*P*)H)-oxidase, myeloperoxidase, xanthine oxidase, and uncoupled nitric oxide synthase (Miller, 2014; Valavanidis, Fiotakis, & Vlachogianni, 2008). A tiered approach to particle toxicity has been formulated for the respiratory effects of inhaled nanoparticles, whereby successive antioxidant defences are depleted (e.g. the lung lining fluid, followed by the epithelial cell defences) eventually leading to insurmountable changes to cell function inducing oxidative stress and changes in cell function (Nel, Xia, Madler, & Li, 2006). A similar scenario is likely to occur in other organs systems following persistent transmission of the signal to that organ (see below). Indeed, a meta-analysis found clear associations between PM exposure of levels of oxidatively-modified lipids and DNA in the blood (Moller & Loft, 2010). Oxidative stress also plays specific roles in the cardiovascular system, one of which is the loss of endothelial cell function. Nitric oxide (NO) is a key mediator within the vasculature, opposing vascular contractility, dampening down the proliferative response in the smooth muscle, inhibiting platelet aggregation and regulating circulating inflammatory cells. Particulates such as DEP have a high capacity to generate the free radical superoxide, which scavenges NO (Miller et al., 2009). The resultant loss of NO bioavailability promotes vasoconstriction, vascular remodelling, platelet aggregation and interaction of the vasculature with inflammatory cells. Thus, the oxidative effects of PM have the capacity to promote numerous steps in the pathophysiology of different cardiovascular diseases.

Inflammation goes hand-in-hand with oxidative stress for many disease processes, but it is also a key pathway in the biological effects of inhaled particles. PM within the lung is readily phagocytosed by alveolar macrophages, presumably as an attempt to defend against the invading xenobiotic. The overload of these cells by large numbers of bio-persistent particulates containing pro-inflammatory chemical constituents readily leads to over-activation of the macrophage triggering an inflammatory response (Stone et al., 2017). The inflammation resulting from neutrophil and macrophage activation, leads to recruitment of additional inflammatory cells to the alveoli, which can amply the inflammation to an extent that causes injury to the surrounding tissue, potentially leading to fibrosis and loss of pulmonary function. PM exposure also promotes inflammation in the cardiovascular system (Hiraiwa & van Eeden, 2013; Liu et al., 2019). A combination of pro-inflammatory changes in endothelial cell phenotype (Shaw et al., 2011) and over-activation of circulating leucocytes (Yatera et al., 2008) leads to associations between the two cell types that are representative of key processes in the initiation of atherosclerosis. Similarly, PM exposure can exacerbate several later stages of different cardiovascular diseases through inflammation, from increased recruitment of inflammatory cells to myocardial infarction, promoting inflammation within atherosclerotic plaques and through synergies with platelets and coagulation factors to increase the risk of thrombosis (Hadei & Naddafi, 2020). The complex molecular mechanisms by which oxidative and inflammatory actions of PM could promote cardiovascular disease are well described in a recent review (Rao, Zhong, Brook, & Rajagopalan, 2018).

**Fig. 2:**
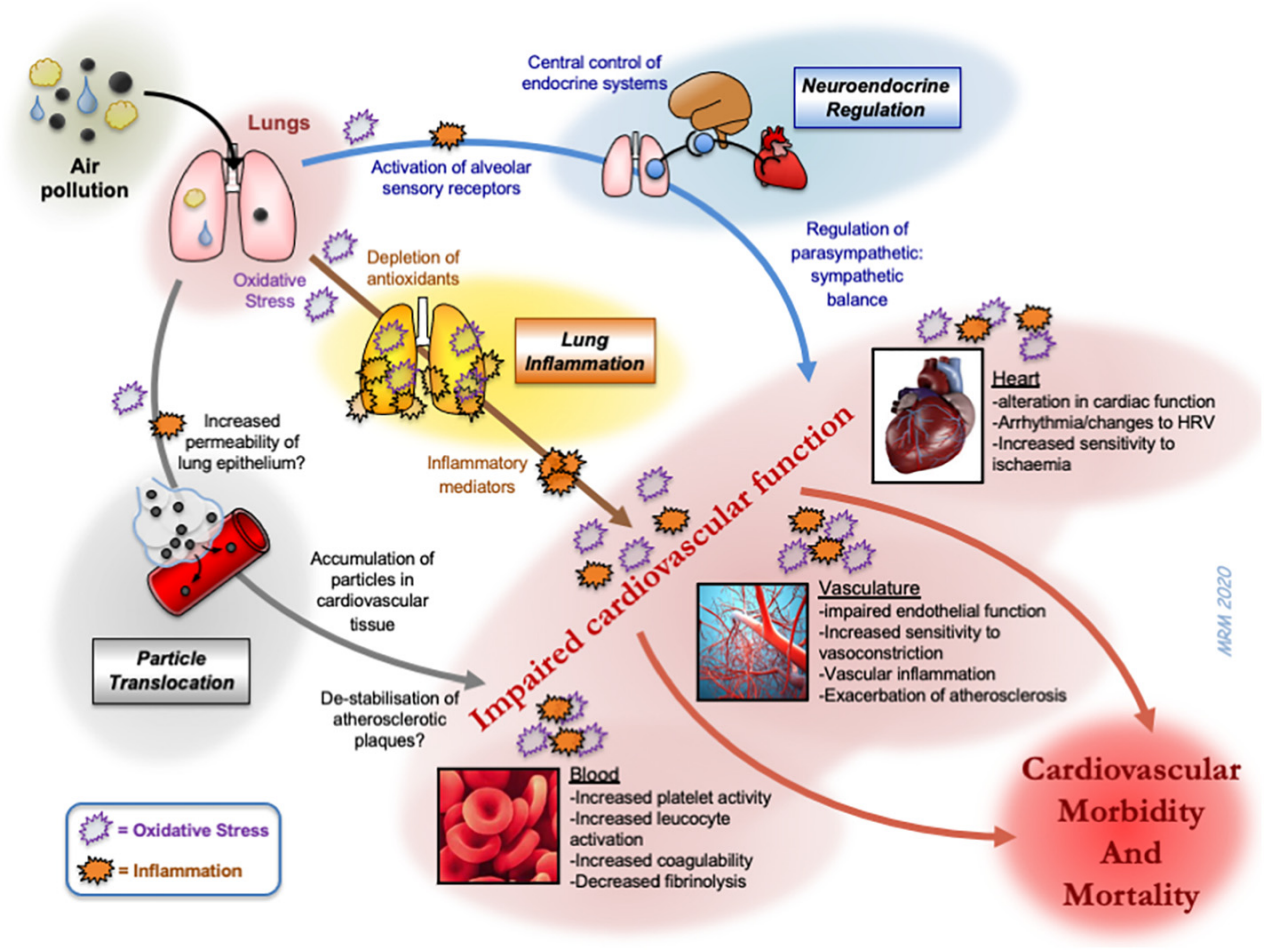
Biological pathways through which inhaled pollutants could induce cardiovascular morbidity and mortality. Three main hypotheses have been proposed for the means by which inhaled particles have actions on the cardiovascular system. These include: 1) the passage of biological mediators (e.g. inflammatory cytokines) from the lung into the circulation; 2) the passage of particles (or chemicals eluting from particles) into the circulation to directly impair cardiovascular function; 3) activation of alveoli sensory receptors leading to triggering of neural afferents which can alter the activity of the autonomic nervous system. Oxidative stress is a common feature at multiple stages of the different pathways. The diagram also highlights that urban PM exerts many pathophysiological changes on different aspects of the cardiovascular system that ultimately increases cardiovascular morbidity and mortality. HRV = heart rate variability. Adapted from (Niemann et al., 2017).

One current area of mechanistic research in this field is the means by which the pulmonary actions of particulates are transferred into actions on the cardiovascular system. Several pathways have been hypothesised (see Miller & Newby, 2020 for further details; Fig. 2). Briefly, the inflammatory response induced by inhaled PM may ‘spill-over’ into the circulation, the mediators of which then induce a systemic response and indirectly affect the cardiovascular system (Seaton, MacNee, Donaldson, & Godden, 1995). Alternatively, inhaled PM (or the inflammation/oxidative stress induced by it) may stimulate sensory receptors on the alveolar surface that leads to changes in cardiac function via alterations in autonomic regulation (Perez, Hazari, & Farraj, 2015) or central-control of endocrine factors (Kodavanti, 2016). Lastly, the smallest ultrafine particles may be sufficiently small to cross the alveolar-capillary barrier themselves and gain access to the blood whereby they may directly interact with the cardiovascular system, or other organs (Miller et al., 2017; Oberdorster et al., 2002). The subtleties of each of these pathways are still being defined, but together these processes have the means to encompass the multiple actions of inhaled PM on cardiovascular function. The relative contribution of these different routes is likely to impact the effectiveness of different agents that could be used to prevent the actions of PM.

## 5 Pharmacological-inhibition of the cardiovascular actions of PM

Surprisingly, there has been relatively little attention given to pharmacological interventions that could block the cardiovascular effects of inhaled PM. A number of epidemiological studies have adjusted for patient medication as confounders in their analysis, although this has not been investigated as a primary aim in large scale studies or meta-analyses. Given the prominent role of oxidative stress, pharmacological approaches to prevent or reverse the effects of air pollution have centred on compounds with antioxidant properties (Barthelemy, Sanchez, Miller, & Khreis, 2020; Peter et al., 2015; Romieu, Castro-Giner, Kunzli, & Sunyer, 2008). A recent review discussed the use of antioxidants to protect against the pulmonary effects of air pollution (Whyand, Hurst, Beckles, & Caplin, 2018). The current review focuses on the use of pharmacological agents on the cardiovascular endpoints affected by PM exposure.

### 5.1 Cardiovascular mortality and morbidity

While a detailed discussion of large cohort epidemiological studies looking at the effects of air pollution are beyond the scope of this review, it is worth mentioning selected studies that have explored the effect of antioxidant-rich diets or supplements on gross cardiovascular endpoints such as mortality rates, or morbidity via hospital admissions and acute cardiovascular events (see also Barthelemy et al., 2020).

A US cohort of over 500,000 adults (ages 50–71) found that long-term exposure (>10 years) to the air pollutants NO_2_ and PM_2.5_ was associated with an increased mortality from cardiovascular causes (e.g. ischaemic heart disease, cerebrovascular disease and cardiac arrest) (Lim et al., 2019). Using participant diaries an “alternative Mediterranean diet” score was formulated with higher scores (e.g. from fruit and vegetable intake, oil-rich fish) representing those with higher antioxidant intake. Participants with greater adherence to the Mediterranean diet were shown to have lower rates of pollutant-attributed cardiovascular mortality. In ~350,000 European subjects, Beelen et al. found associations between long-term exposure to PM_2.5_ and mortality from cerebrovascular causes, but not other cardiovascular causes of mortality (although air pollution levels in this study were generally low and medication use was not fully addressed) (Beelen et al., 2014). Adjustment for dietary factors (fruit and vegetable intake) did not influence the association between PM_2.5_ and cerebrovascular mortality. A previous study from the same group found that ‘natural-cause’ mortality was higher with exposure to black smoke (a surrogate for combustion-derived PM) (Beelen et al., 2008). This effect was greater in those with low fruit consumption, although the effect did not reach statistical significance.

In regards to cardiovascular morbidity, PM_2.5_ has been associated with an increased risk in the incidence of coronary atherosclerosis and myocardial infarction in Ohio, USA (Hartiala et al., 2016). These associations were not affected by adjustment for statin therapy. In a US study of nurses, the risk of myocardial infarction was found to be inversely associated with distance of residential address from a major road (Hart, Rimm, Rexrode, & Laden, 2013). While the association was not changed by adjustment with an index of healthy diet (albeit dietary information was only available on a 4-year basis), there was a slight attenuation in associations with myocardial infarction when restricting analysis to categories that were closest and furthest away from the roads.

### 5.2 Cardiac effects

#### 5.2.1 Heart rate variability

Exposure to air pollution is associated with changes to the rhythm and contractility of the heart. Many epidemiological studies have made use of the Holter monitoring of electrocardiogram (ECG) recordings as a non-invasive means of assessing the cardiac effects of air pollution (Buteau & Goldberg, 2016). Heart rate variability (HRV) is a set of parameters derived from detailed analysis of the regularity of the ECG. These parameters are indicative of the modulation of the electrical activity of the heart, in particular its regulation by the autonomic nervous system. For most HRV parameters, a reduction would confer a greater risk of developing cardiovascular conditions (at a population level), with reduced HRV linked to increased mortality from sudden death and ventricular arrhythmia in both healthy and diseased individuals (Kleiger, Miller, Bigger Jr., & Moss, 1987; Lahiri, Kannankeril, & Goldberger, 2008). While there is a great deal of inconsistency between parameters and individual studies, both gases and PM in air pollution reduce heart rate variability in a manner that is indicative of increased sympathetic drive and decreased parasympathetic input (Buteau & Goldberg, 2016).

##### 5.2.1.1 Antioxidants

A number of studies have considered whether antioxidant-rich diets can ameliorate the effects of air pollution on HRV. Romieu and colleagues provided participants (>60 years old) with either a fish oil or soy oil (control) supplement for 6-months (Holguin et al., 2005; Romieu et al., 2005). PM_2.5_ exposure was associated with detrimental reduction in selected HRV parameters. These effects were significantly decreased (i.e. ‘improvement’ in HRV) in those taking the fish oil supplements, whereas soy oil supplements had lesser or no benefits. PM_2.5_ was associated with detrimental changes in HRV in elderly men with genetic deficiencies in methionine pathways, the effects of which were lessened in those with higher intakes of methionine or vitamins B6 or B12 (Baccarelli et al., 2008). Chamber studies, where volunteers are asked to inhale specific pollutants under controlled conditions for short periods (usually 1–2 h), have also been used to investigate if antioxidant supplements prevent the effects of pollution on HRV (Tong et al., 2012). Four weeks of oral omega-3 fatty acid supplements attenuated CAPs-induced reductions in high to low frequency ratio metrics and elevations in normalized low-frequency HRV; a benefit that was not evident in the control group (olive oil supplements). Additionally, this study also found that the fish-oil supplements improved lipid profiles in these volunteers. Animal studies also demonstrate beneficial effects of antioxidants on the cardiac parameters following PM exposure. For example, HRV changes in response to pulmonary administration of urban PM to rats were prevented by the antioxidant *N*-acetylcysteine (NAC) (Rhoden, Wellenius, Ghelfi, Lawrence, & Gonzalez-Flecha, 2005). Additionally, the cardiac oxidative stress induced by the PM was inhibited by beta-blockers. These results highlight the difficulty epidemiological studies face in establishing the cardiac effects of PM in patients that are often taking on multiple medications.

##### 5.2.1.2 Beta-blockers

HRV is principally derived from regulation of the heart via the autonomic nervous system and, accordingly, beta-blockers can prevent the changes in HRV associated with air pollution. Using a combination of personal (measured on individuals using a portable device) and ambient (use of stationary monitoring networks) air pollution, outdoor PM_2.5_ (particularly that associated with traffic) was linked to detrimental effects on HRV parameters, but only in patients that were not taking beta-blockers (de Hartog et al., 2009). The same group measured S-T segment depression (a region of the ECG that can indicate ischaemic stress in coronary arteries) in patients with stable coronary artery disease while exercising in the city of Helsinki, Finland (Pekkanen et al., 2002). Carbon monoxide, NO_2_, PM_2.5_, particle number (used as a surrogate for ultrafine PM), but not the larger coarse PM, was associated with an increased prevalence of S-T segment depression. The associations were stronger in those not taking beta-blockers, indicating that these effects may be reflective of autonomic regulation of the heart.

##### 5.2.1.3 Statins

Statins are now a ubiquitous medicine used to lower blood cholesterol, although their pleotropic actions (e.g. anti-oxidant, anti-inflammatory) are well recognised. In middle-aged women, statins were found to eliminate associations between PM_2.5_ and blood levels of C-reactive protein (a marker of inflammation/acute phase response) (Ostro et al., 2014). In relation to cardiac parameters, statins were shown to completely prevent the effects of PM_2.5_ on high-frequency HRV parameters in individuals that were null for the glutathione-S-transferase M1 (GSTM1) allele (making these individuals more susceptible to the effects of oxidative stress) (Schwartz et al., 2005). The investigators propose that reductions in oxidative stress were partially to account for the beneficial effects.

##### 5.2.1.4 Angiotensin antagonists

Long-term exposure to traffic-related PM_10_ was associated with HRV in middle-aged to elderly subjects in those taking angiotensin converting enzyme (ACE) inhibitor medication, but not those who were not (Adam et al., 2012). This reason for this is not presently clear, and deserves further investigation.

#### 5.2.2 Arrhythmia

Air pollution has also been shown to induce distinct periods of arrhythmia in epidemiological studies and animal models of drug-induced arrhythmia (Carll et al., 2010; Carll et al., 2013; Carll et al., 2015). A number of laboratory studies have addressed the role of oxidative stress in arrhythmia using antioxidant compounds. Direct addition of DEP to cultured cardiomyocytes reduced contractile function, an effect that could be partially prevented by free radical scavengers (NAC or tiron) or inhibitors of cellular sources of free radicals (oxypurinol to inhibit xanthine oxidase; apocyanin to inhibit NAD(*P*)H oxidase) (Gorr et al., 2015). The direct cytotoxicity or apoptotic actions of various PM on cardiomyocytes can be inhibited by NAC or diemethylthiourea (a scavenger of hydroxyl radicals and hydrogen peroxide) (Kim et al., 2012; Knuckles et al., 2013). Pulmonary exposure of rats to DEP prolonged the cardiac action potential and induced premature ventricular contractions (Kim et al., 2012). These effects could be prevented by pre-treatment with NAC. Robertson et al. used pulmonary instillation of DEP in a rat model of cardiac ischaemia induced by coronary artery ligation (Robertson et al., 2014). Ligation was accompanied by long-lasting cardiac arrhythmias, which were associated with high levels of mortality. These effects were reduced by co-administration of a β_1_ receptor antagonist (metoprolol) or blockade of the alveolar sensory receptors with a vanilloid receptor (transient receptor potential cation channel V1; TRPV1) antagonist.

#### 5.2.3 Myocardial infarction and heart failure (animal models)

Long-term exposure to air pollution has been associated with an increased incidence of heart failure (Shah et al., 2013), with loss of the contractility of ventricular cardiomyocytes and the development of compensatory hypertrophy (Wold et al., 2012). Coronary artery ligation in rats has been used to induce myocardial infarction (Robertson et al., 2014). After a 45 min period of ischaemia, blood flow was restored, and various staining methods were used to detect viable, ischaemic and necrotic areas of the cardiac wall. Pulmonary instillation of DEP prior to injury caused a 3-fold increase in the extent of myocardial infarction. These effects were reduced by co-administration of a β_1_receptor antagonist or blockade of alveolar TRPV1 receptors. These findings nicely demonstrated that DEP can precondition the heart to increase susceptibility to ischaemic damage through sensory afferents via autonomic innervation. A separate group demonstrated a beneficial effect of combined β_1_-antagonist and β_2_-agonist therapy on preventing the cardiac dysfunction caused by PM_2.5_ exposure in a rat model of acute myocardial infarction (Gao et al., 2014). A similar model was used to demonstrate that urban PM_10_ decreased endothelial nitric oxide synthase (eNOS) and antioxidant enzyme expression in cardiac tissue, together with increased expression of pro-inflammatory inducible nitric oxide synthase (iNOS) (Dianat, Radmanesh, Badavi, Mard, & Goudarzi, 2016). The antioxidant vanillic acid reduced the effects of PM_10_ on cardiac antioxidant levels, and partially improved mitochondrial disturbances and cardiac performance. Repeated exposure to PM_2.5_ also promoted cardiac oxidative stress and inflammation in otherwise healthy rats (Zeng et al., 2018). Supplementation with selenium-rich yeast dose-dependently inhibited these effects. Finally, nootkatone (an anti-inflammatory and anti-oxidative compound found in grapefruit extract) inhibited oxidative stress in the hearts of mice exposed to DEP (Nemmar, Al-Salam, Beegam, Yuvaraju, & Ali, 2018).

In light of evidence linking air pollution to heart failure, an intricate study by Rajagopalan's group explored the effects of PM on cardiac remodelling (Ying et al., 2009). Remodelling in rats was induced by a 14-day infusion of angiotensin II, following a 12-week inhalation of CAPs or filtered air. CAPs increased cardiac remodelling and collagen deposition, an effect that could be prevented by co-infusion of fasudil (a Rho kinase inhibitor). Together with detailed molecular exploration of guanine exchange factors, their findings indicated that RhoA/Rho kinase pathways were key to the cardiac remodelling.

### 5.3 Vascular system

#### 5.3.1 Endothelial function

**Fig. 3:**
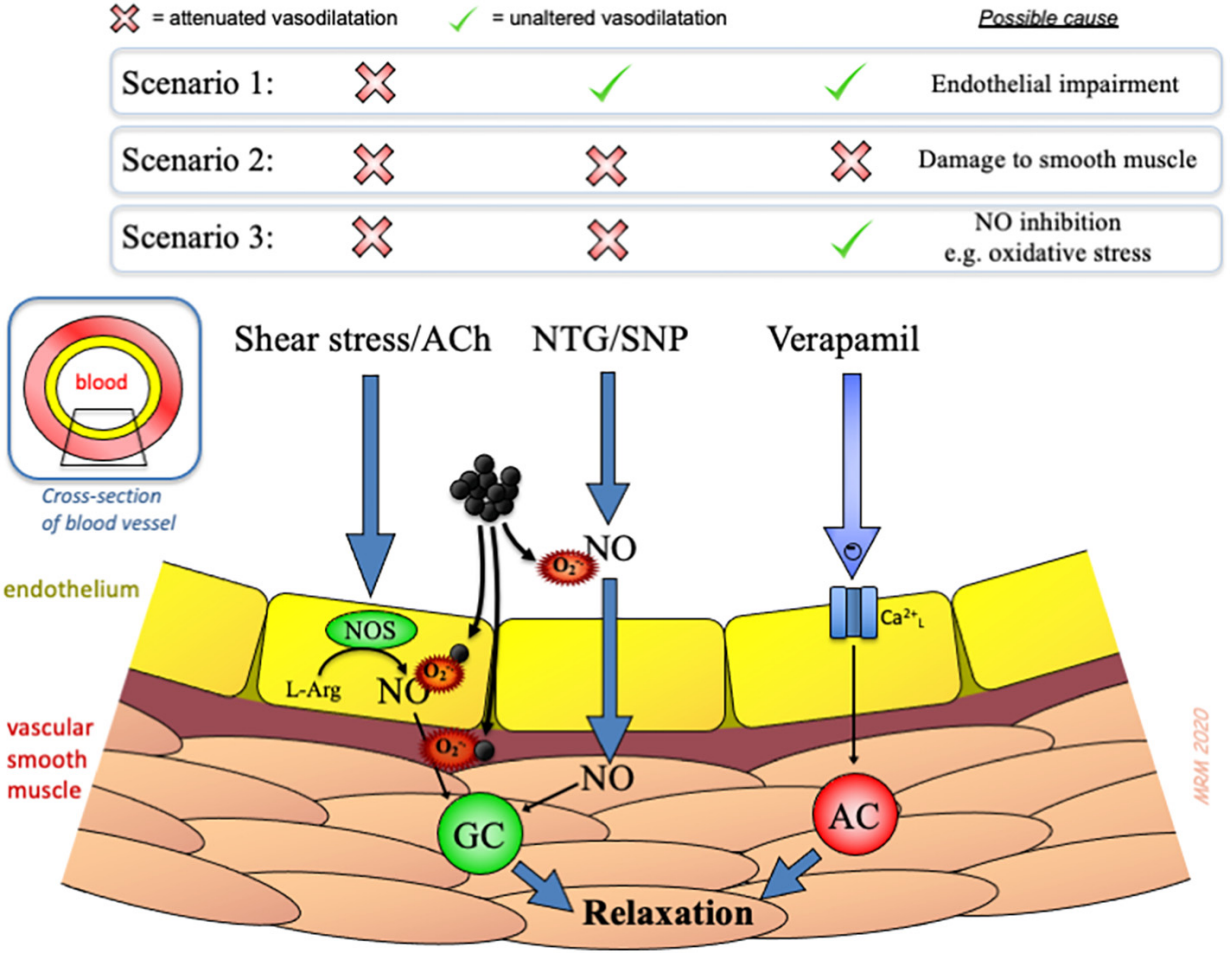
Pharmacological approaches to assessing the mechanisms of the impaired vasodilator responses following exposure to pollutants. A combination of different stimuli and drug infusions can be used to explore the mechanisms by which vasodilatation is impaired (see example scenarios). Changes in blood flow and infusion of drugs such as acetylcholine (ACh) and bradykinin (BK) stimulate endothelial cells to synthesise nitric oxide (NO). NO diffuses to the smooth muscle to activate guanylate cyclase (GC) which ultimately induces relaxation of the vascular smooth muscle and dilatation of the blood vessel. Drugs such as nitroglycerin (NTG or glyceryl trinitrate) and sodium nitroprusside (SNP) act independently of the endothelium to generate NO from their molecular structure. Drugs such as verapamil (and isoprenaline in rodent models) activate receptors on vascular smooth muscle cells to induce vasodilatation independently of NO. Exposure to PM tends to inhibit pathways involving NO, but not NO-independent pathways. This pattern suggests that oxidative stress is a prominent mechanism of action, due to the scavenging of NO by superoxide free radicals (O_2_^-.^). Other abbreviations: AC = adenylate cyclase, Ca^2+^_L_ = L-type calcium channel, NOS = nitric oxide synthase. Reproduced from (Miller, 2020).

Inhalation of air pollutants promotes contractility of both the pulmonary circulation and systemic vascular beds (Moller et al., 2011). Numerous studies, both epidemiological (Brook et al., 2010; Newby et al., 2015) and controlled exposure to pollutants (Brook et al., 2002; Mills et al., 2009), have shown that inhaled PM leads to a change in vascular tone to promote vasoconstriction and decrease vasodilator responses. Endothelial dysfunction is a key characteristic by which vasodilator capacity is lost, with oxidative stress in particular being a prominent mechanism (Miller, 2020; Miller et al., 2012). The use of pharmacological agents to induce vasodilation through different mechanisms (Fig. 3) has been used to show that PM exposure impairs vasodilators acting through the NO pathway (both endothelial-dependent dilators and endothelial-independent NO-donor drugs) which is suggestive of scavenging of this essential cardiovascular messenger by superoxide (Mills et al., 2009).

##### 5.3.1.1 Antioxidant agents

Similar to the HRV studies, the ability of antioxidant-rich oil supplements to ameliorate the effects of air pollution have been considered using flow-mediated (endothelium-dependent) dilatation of the brachial artery. In middle-aged volunteers, controlled exposure of CAPs led to a decrease in flow-mediated dilatation (Tong et al., 2015). Counter-intuitively, the fish-oil supplements did not have a beneficial effect on this response, whereas an olive oil supplement (used as control compound in previous studies by the investigators) did. The authors speculate that the anti-inflammatory and anti-oxidative actions of oleic acid in the olive oil could account for these beneficial effects, although it is not immediately clear why these results should differ from those by the same authors which show other cardiovascular benefits of fish oil but not olive oil. Another controlled exposure study, this time to diesel exhaust, found that an antioxidant regime (Vitamin C combined with NAC) increased the vasoconstriction caused by diesel exhaust, rather than preventing it (Sack et al., 2016). While the authors discuss a number of possible explanations for the unexpected findings, they conclude that further work would be needed to reach a satisfactory explanation.

Organ bath myography techniques have been used to show that segments of rat coronary arteries exhibited impaired endothelium-dependent relaxation after inhalation of diesel exhaust (Cherng et al., 2011). Addition of superoxide dismutase (SOD) to the arteries reversed the effects of DEP, suggesting an on-going production of superoxide in these arteries leading to loss of NO bioavailability. Direct application of isolated rodent arteries with DEP has been used to explore mechanistic pathways of nanoparticles that could potentially translocate from the lung into the circulation. The results parallel those of in vivo exposures, in that both endothelium-dependent and NO-dependent vasodilators are inhibited by diesel exhaust particle exposure (Ikeda et al., 1995; Labranche et al., 2012; Miller et al., 2009). In these studies, SOD partially prevented the vascular impairment, again demonstrating a role for superoxide free radical. Aortic rings taken from the PM_2.5_ exposed mice exhibited a greater contraction to phenylephrine and reduced dilatation to acetylcholine, as well as potentiation of the response to a Rho-kinase inhibitor (Sun et al., 2008; Ying et al., 2009). Apocyanin or nitric oxide synthase (NOS; the enzymatic source of NO) inhibition partially prevented the effect of PM_2.5_, indicating a likely role for superoxide generation from NAD(*P*)H-oxidase and NOS uncoupling, respectively (Sun et al., 2008). NAC inhibited the effects of ultrafine particles in PM_2.5_ after direct addition to isolated arteries. NAC and other SOD mimetics have been shown to attenuate the effects of various sources of PM in endothelial cells, e.g. inflammation, down-regulation of NOS, tight-junction degradation (Du et al., 2013; Rui, Guan, Zhang, Zhang, & Ding, 2016; Sharma et al., 2019; Tseng et al., 2015; Tseng, Wang, Chang, Chang, & Chao, 2015). Additionally, inhibitors of inflammatory pathways, myosin light chain kinase and calcium channel (Ca^2+^_L_) blockers can also reverse the effects of PM on cultured endothelial cells (Rui et al., 2016; Tzeng, Yang, Ueng, Lin-Shiau, & Liu, 2003).

Urban PM can generate superoxide through a number of different pathways to induce a combined insult to endothelial function (Miller, 2014; Miller et al., 2012). These include redox reactions and superoxide formation from the particle surface and from interaction with inflammatory cells, but also from stimulation of cellular enzymes in other cell types. A role for superoxide generation from NAD(*P*)H oxidase has been implicated in the vascular dysfunction in rats after inhalation of urban PM_2.5_ (Kampfrath et al., 2011) or DEP (Labranche et al., 2012). Accordingly, the NAD(*P*)H oxidase inhibitor, apocyanin, restored NO generation from endothelial cells treated with ultrafine particles (Du et al., 2013; Sun et al., 2008).

De-regulation of eNOS is another potential mechanism of endothelial dysfunction. Two weeks exposure of rats to PM_2.5_ impaired pulmonary artery vasodilation and decreased eNOS expression (Davel et al., 2012). Sub-chronic exposure to PM_2.5_ leads to depletion of the co-factors needed for the NO-generation from eNOS (Sun et al., 2008). Furthermore, the use of NOS inhibitors or additional of NOS co-factors can prevent the impairment induced by PM (Sun et al., 2008). The antioxidant Tempol has also been shown to prevent the vascular insulin resistance induced by CAPs via alterations in eNOS phosphorylation (Haberzettl, O'Toole, Bhatnagar, & Conklin, 2016). These findings demonstrate that NOS uncoupling, causing counter-production of superoxide from NOS instead of NO, is an important mechanism by which PM causes vascular impairment.

##### 5.3.1.2 Angiotensin and endothelin pathways

In addition to loss of vasodilator pathways, exposure to PM can upregulate vasoconstrictor mediators. Angiotensin II is a potent endocrine vasoconstrictor molecule. Losartan (an angiotensin II type-1 receptor antagonist) could reverse effects of PM_2.5_ on senescence in cultured endothelial cells and the impaired endothelial dilatation in isolated coronary arteries (Sharma et al., 2019). Similarly, in isolated pulmonary arteries, the pro-constrictor effect of urban PM could be prevented by losartan (Li, Carter, Dailey, & Huang, 2005). Endothelin-1 (ET-1) is another potent vasoconstrictor mediator, known for paracrine and endocrine actions on endothelial and vascular smooth muscle cells, as well as the ability to exacerbate inflammation and oxidative stress in the vasculature. Two studies have shown that antioxidant-rich fish oil supplements have been shown to reduce the levels of circulating vasoconstrictor ET-1 that are associated with ambient PM_2.5_ exposure (Lin et al., 2019) or controlled exposure to CAPs (Tong et al., 2015).

The role of endothelin pathways was also explored in-depth in healthy volunteers after controlled exposure to diesel exhaust (Langrish et al., 2009). Forearm plethsymography was used to show that diesel exhaust decreased endothelial- and NO-dependent vasodilation. Diesel exhaust itself did not increase levels of ET-1 in the blood, however, infusion of ET-1 led to a disproportionately large vasoconstrictor response following diesel exhaust inhalation. Through infusion of combinations of ET_A_ and ET_B_ receptor antagonists, it was determined that the pro-constrictor effect of DE was not necessarily due to up-regulation of ET-1 receptor levels, but more likely to be due to an exaggerated response to vasoconstriction due to the loss of the countering dilatation of NO. In support of this finding, a rat study using NOS and endothelin receptor inhibitors suggested that the pro-constrictor effect of ET-1 in diesel exhaust-exposed animals was due to a combination of both loss of NOS and inhibition of endothelial ET_B_ receptors (Cherng et al., 2011).

#### 5.3.2 Blood pressure

Exposure to air pollution affects not only on the pulmonary blood vessels, but also conductance and resistance arteries in the systemic circulation. The pro-constrictor/anti-vasodilator effects of air pollution in resistance arteries can be accompanied by elevations in blood pressure (“hypertension”) (Brook & Rajagopalan, 2009; Yang et al., 2018). While these increases are often small (e.g. 1–5 mmHg per interquartile increase in pollutant), high blood pressure is a major risk factor for cardiovascular disease, and even small elevations in blood pressure across a population would be expected to be associated with substantial cardiovascular morbidity.

Several studies have addressed the ability to antioxidants to limit the hypertensive effects of air pollution. Brook et al. found that a 2-h exposure of PM_2.5_ and ozone in healthy volunteers led to an increase in blood pressure (2.5–4 mmHg), without changing brachial artery diameter (a conductance vessel) (Brook et al., 2009). The endothelin antagonist, bosentan, caused a marginal blunting of the hypertensive response, whereas responses in the vitamin C group were not significantly different from the placebo. Recently, cardiovascular performance was assessed in fit individuals carrying out a strenuous exercise test in polluted versus non-polluted regions of the city of Tunisia (Boussetta, Abedelmalek, Khouloud, Ben Anes, & Souissi, 2019). Exercising in pollution was associated with higher heart rate and systolic blood pressure. Interestingly, consuming a half litre of red-orange juice (containing high levels of flavanones and vitamin C) prior to the exercise blunted the cardiovascular effects of pollution.

In a detailed mechanistic study, Sun et al. performed a 10-week exposure to PM_2.5_ in a rat model of hypertension induced by infusion of angiotensin-II (Sun et al., 2008). There was substantially greater blood pressure (up to 30 mmHg) between the PM_2.5_ versus filtered air groups. In a spontaneously hypertensive rat model, while in vivo PM exposure did not affect blood pressure itself, isolated blood vessels from exposed rats exhibited an attenuated ex vivo relaxation to endothelial dependent vasodilators via a mechanism that involved NAD(*P*)H oxidase (Labranche et al., 2012). Finally, thymoquinone, a compound from Nigella seeds with anti-inflammatory and antioxidant activity, inhibited the increased systolic blood pressure induced by acute exposure to DEP in mice (Nemmar et al., 2011).

#### 5.3.3 Atherosclerosis

Loss of endothelial function is a hallmark of the early stages of atherosclerosis, leading to accumulation of inflammatory cells and lipids in the vascular wall of arteries. In particular, low density lipoprotein (LDL) in the blood can become oxidised (oxLDL) and preferentially taken-up by macrophages leading to the growth of lipid-rich plaques that characterise atherosclerosis. The erosion or rupture of advanced plaques may induce thrombosis which can occlude the artery triggering a cardiovascular event such as a heart attack or stroke. Air pollution has been associated with all these different stages of atherothrombotic disease, including loss of endothelial function, pro-inflammatory changes in endothelial cells, oxidation of circulating lipoproteins, growth of atherosclerotic lesions, indicators of plaque vulnerability and increased thrombogenicity of the blood (see Miller et al., 2012; Moller et al., 2011). These mechanistic findings underpin epidemiological observations that air pollution is associated with an increased incidence of atherosclerosis, myocardial infarction and stroke (Brook et al., 2010).

Because the development of atherosclerosis occurs over many decades in humans, there are limited clinical studies on the effects of pollution on atherosclerosis. However, blood lipid profiling has been used to consider one facet of the pro-atherosclerotic effects of air pollution. For example, 4 weeks prior to inhalation of CAPs in middle-aged volunteers led to increases in blood triglycerides and very low-density lipoprotein, and a trend towards increased total cholesterol (Tong et al., 2012). These changes were evident in volunteers taking olive oil supplements, but not those fish oil supplements, suggesting that the anti-oxidant properties of the fish oil supplements could off-set the effects of PM exposure on lipids. Neither of the supplements had particularly striking effects on blood lipids prior to CAPs exposure. Adhesion molecules play a role in tethering inflammatory cells to the vascular wall in the early stages of atherogenesis. Alexeeff and colleagues found associations between black carbon exposure (a constituent of PM often used as a surrogate for traffic-derived PM or other combustion sources) and blood levels of the adhesion molecule intercellular adhesion molecule-1 (ICAM-1) (and a trend for vascular cell adhesion molecule-1 (VCAM-1)) in elderly men (Alexeeff et al., 2011). Associations were greater in those who were diabetic, whereas the associations were not observed in individuals taking statins. Separate studies in elderly (Alexeeff et al., 2011) or diabetic individuals (O'Neill et al., 2007) also found stronger associations between PM_2.5_ and BC exposure and levels of ICAM-1, VCAM-1 and von Willibrand factor (vWF) in those not taking statins, compared to those that were.

Cell culture studies have also addressed some of the early stages of atherosclerosis. Despite the pro-oxidative nature of DEP, addition of DEP directly to cultured endothelial cells in vitro had limited effects on cell phenotype unless very high concentrations were used (Shaw et al., 2011). However, if DEP was first incubated with monocyte-derived macrophages, followed by treatment of the endothelial cells with the mediators released from the macrophages, there was a marked activation of endothelial cells in terms of expression of pro-inflammatory adhesion molecules and release of cytokines. These effects could be partially inhibited with the tumour necrosis factor alpha (TNF-α) binding agent, etanercept, suggesting that TNF-α released from DEP-stimulated macrophages is an important regulator of DEP-induced endothelial dysfunction.

Transgenic mice, such as apolipoprotein-E (ApoE) knockout mice, have been used to elaborate on the mechanisms underlying the development of atherosclerosis. These mice cannot clear cholesterol from the blood effectively, leading them to develop high levels of plasma cholesterol and rapid formation of atherosclerotic plaques within 5–10 weeks of high fat feeding. There is a large body of research using these murine models to demonstrate that air pollution promotes both the growth and development of atherosclerotic plaques, and potentially their vulnerability to rupture (Moller et al., 2011). There are surprisingly few studies using pharmacological agents to prevent the pro-atherosclerotic effects of air pollution in atherosclerosis-prone mice, possibly in part due to the complex multifaceted nature of underlying pathways. For example, a four-month CAPs inhalation study in fat-fed ApoE knockout mice led to increased lipid and macrophage content of atherosclerotic plaques, as well as an increase in NAD(*P*)H oxidase expression (Ying et al., 2009). Vascular dysfunction was also observed, including an attenuated contractile response to phenylephrine which could be restored by a soluble guanylate cyclase (sGC) inhibitor. The investigators speculate that sGC may have been upregulated in vascular smooth muscle cells to compensate for loss of NO bioavailability. Lastly, while free radical scavenging compounds have not been tested in pollutant-treated atherosclerotic mice, selenium supplementation, was found to reduce both oxidative stress and VCAM-1 levels in healthy rats, which could ameliorate the early stages of atherosclerosis (Zeng et al., 2018). Together these studies support the theory that oxidative stress is a key pathway in the pro-atherosclerotic effects of PM exposure (Miller et al., 2012; Miller et al., 2013).

### 5.4 Haemostasis

#### 5.4.1 Biomarkers of thrombogenicity

Exposure to air pollution has been shown alter a number of different biomarkers that are associated with increased thrombogenecity of the blood. These include fibrinogen, tissue factor, vWF, P-selectin, as well as decreases in the activity of fibrinolytic mediators that mediate clot breakdown (Delfino et al., 2009; Franchini, Guida, Tufano, & Coppola, 2012; Robertson & Miller, 2018). Despite the involvement of multiple targetable mechanisms, there is a paucity of studies that have used pharmacological approaches to prevent the pro-thrombotic effects of air pollution. Associations between markers of combustion-derived PM and circulating P-selectin were weaker in elderly subjects taking the anti-platelet agent clopidogrel compared to those that were not (Delfino et al., 2009). A few panel studies have investigated fish-oil supplements. Healthy college students in Shanghai, China, given five months of fish oil supplements during the polluted winter season, showed reduced blood fibrinogen and vWF levels compared to the placebo group (sunflower oil), in parallel to improving markers of blood anti-oxidant status and endothelial function (Lin et al., 2019). Endothelial cells release the fibrinolytic molecule tissue plasminogen activator (t-PA) that mediates the breakdown of blood clots. Unexpectedly, controlled exposure to CAPs increased t-PA levels in elderly individuals, possibly as a compensatory mechanism to counter pro-thrombotic pathways (Tong et al., 2015). t-PA release was greater in participants taking olive oil supplements compared to those who were not taking supplements. This suggests that the antioxidant effects of the oil improved endothelial function, although the lack of a similar effect in those taking fish oil supplements was surprising. The study also showed some indications that ACE inhibitors or statins influenced the action of CAPs on D-dimer levels, although the study was not powered to sufficiently address this point. Other studies have also showed that associations between exposure to combustion-derived PM and circulating vWF (as well as markers of inflammation and oxidative stress) are weaker in individuals taking statins compared to those who are not (Delfino et al., 2009; O'Neill et al., 2007).

#### 5.4.2 Thrombosis models

Laboratory studies have the advantage of being able to address the functional process of thrombosis itself rather than biomarkers in isolation. Both urban PM and oil-fly ash PM decreased the time required for blood to clot in vitro (Metassan, Charlton, Routledge, Scott, & Ariens, 2010; Sangani, Soukup, & Ghio, 2010). This effect could be inhibited by complexing iron with deferoxamine or the hydroxyl-radical scavenger mannitol, suggesting that iron in the PM catalysed the production of hydroxyl free radicals leading to increased blood coagulability. In another in vitro assay, Nemmar and colleagues demonstrated that the antioxidant/anti-inflammatory agent, emodin, prevented the increased platelet activation and thrombosis caused by DEP (Nemmar et al., 2015). Furthermore, treatment of cultured endothelial cells with ultrafine PM promoted the ability of these cells to generate active thrombin from its substrate (Snow, Cheng, Wolberg, & Carraway, 2014). This effect was attributed to free radical generation as the thrombin generation could be reversed by SOD and catalase (which breakdown superoxide and hydrogen peroxide, respectively). The effects were also reversed using diphenyleneiodonium (an NAD(*P*)H-oxidase inhibitor) but not rotenone (an inhibitor of mitochondrial-derived free radicals) or allopurinol (a xanthine oxidase inhibitor), identifying NAD(*P*)H oxidase as the cellular source of the free radicals.

Molecular interactions between the vessel wall and blood cells are important physiological determinants of blood clotting. Accordingly, vascular injury models can be used to study thrombosis at the site of injury to better model atherothrombosis in response to endothelial degradation or endothelial damage after surgical injury (e.g. after angioplasty). DEP instillation increased thrombosis after photochemical injury of cerebral arterioles (Nemmar et al., 2015; Nemmar, Al-Salam, Dhanasekaran, Sudhadevi, & Ali, 2009; Nemmar, Subramaniyan, & Ali, 2012). This effect could be inhibited by compounds with antioxidant and anti-inflammatory properties, such as a cysteine pro-drug (Nemmar et al., 2009), dexamethasone (Nemmar, Hoet, Vermylen, Nemery, & Hoylaerts, 2004), curcumin (Nemmar et al., 2012), nootkatone (Nemmar et al., 2018), thymoquinone (Nemmar et al., 2011) or emodin (Nemmar et al., 2015). The same group also used histamine receptor antagonists to implicate mast cells in the pro-thrombotic effects of DEP (Nemmar, Nemery, Hoet, Vermylen, & Hoylaerts, 2003). Finally, β_2_-adrenoreceptor agonists were shown to inhibit the pro-thrombotic effects of PM in mice (Chiarella et al., 2014). The authors postulated that PM stimulates the release of prothrombotic interleukin-6 from macrophages via β_2_ receptors.

## 6 Conclusions

It is now widely recognised that air pollution is associated with considerable cardiovascular morbidity and mortality. Over the last two decades, a wealth of scientific research has elucidated the underlying mechanisms for these associations. While complex, a number of compelling pathways have been formulated that support the associations derived from epidemiological studies, and support a case for causality between air pollution exposure and cardiovascular disease. Nonetheless, further research will be vital in identifying additional key pathways and elucidating the finer details of pathways that are already established. Pharmacological approaches will be a useful tool in achieving these aims.

The current review provides an overview of studies that have used pharmacological agents to prevent, reverse and, in a few cases, augment the cardiovascular effect of air pollution (Table 1). While this review attests to substantial body of high-quality research in this area, the use of pharmacological approaches is still limited in many regards. For example, there are many different constituents of air pollution associated with detrimental health effects, yet almost all of the studies described in this review focus on a select number of pollutants; largely particulate matter (with an urban focus) or controlled exposures to vehicle exhaust, principally diesel exhaust. While this is an observation that can be levelled at the field of air pollution and health in general, it is particularly noticeable here. Secondly, additional studies are needed in human subjects. While antioxidant supplements have been investigated in relation to air pollution to a moderate extent (see below), there is a distinct lack of human studies investigating other therapies. Large cohort databases have been used to determine associations between health parameters and pollutant exposure, and occasionally medication has been considered as a confounding variable. The brief review of the epidemiological studies of this type in the present review reveals a mixed picture. In many cases, stronger associations between air pollutants and cardiovascular parameters are found in patients not taking medication compared to those that are. However, contrary to this, associations derived from larger samples of broader population (i.e. healthy individuals and prescribed patients) often find associations between PM_2.5_ and cardiovascular morbidity that remains after adjustment for medication. A detailed sub-analysis of the effects of individual patient medication, followed by meta-analyses of different cohorts, could yield valuable insight into which medicines interact with the effects of pollution.

**Table 1:**
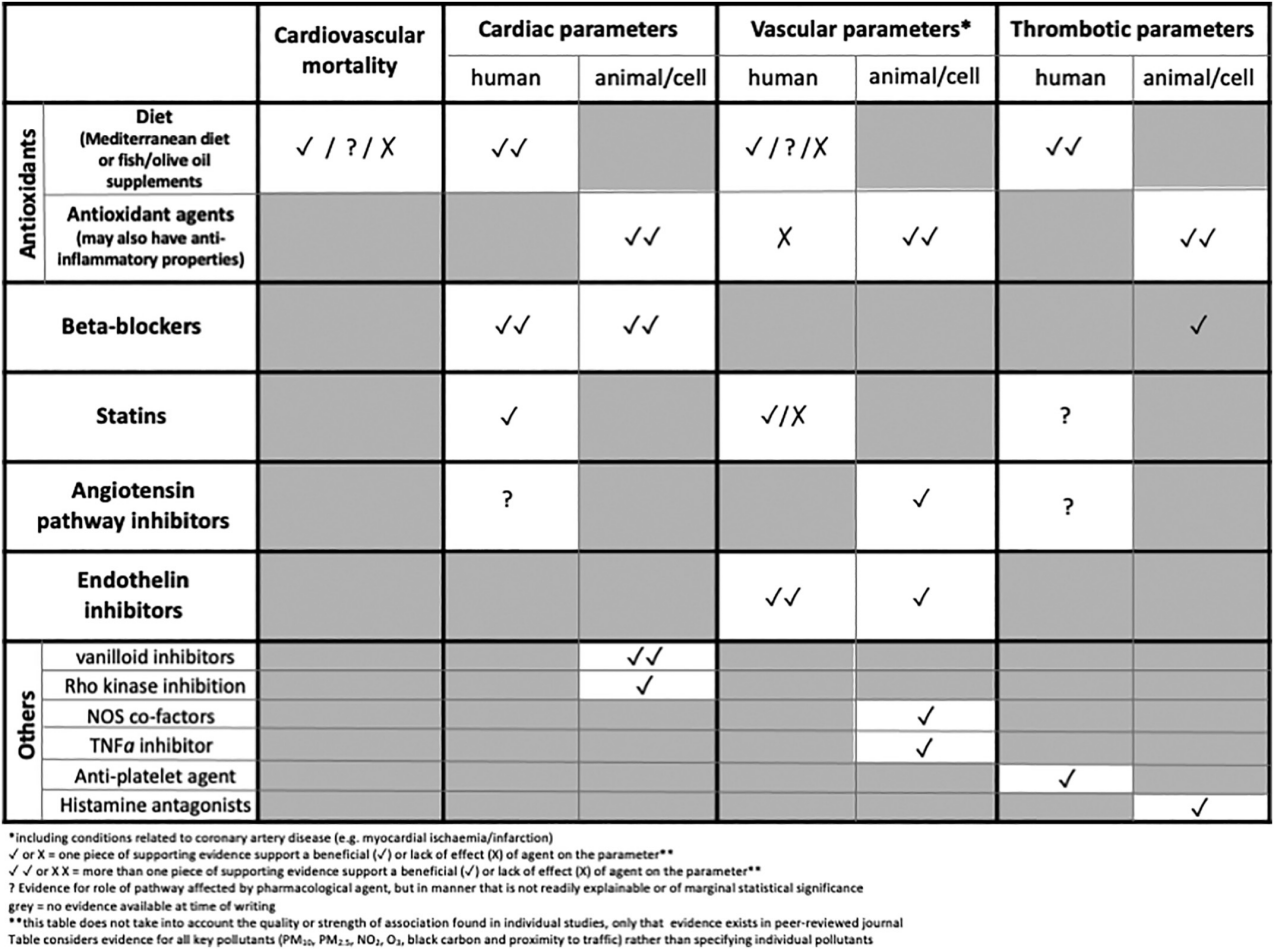
Overview of the number of studies providing evidence for an effect of a pharmacological agent on cardiovascular parameters linked to air pollution exposure.

This summation of the evidence derived from pharmacological approaches to prevent the cardiovascular effects of air pollution highlights the rather limited range of pathways explored. Given the convincing evidence for a role of oxidative stress in many of the pathophysiological actions of air pollution, it is unsurprising that antioxidant agents are the basis of most of the studies described in this review. Studies in humans (both epidemiological and controlled exposures) have made good use antioxidant-rich oil supplements (especially omega-polyunsaturated fish oils) to demonstrate that these supplements have beneficial actions on a range of cardiovascular endpoints in exposed individuals (Barthelemy et al., 2020). In several cases, some clarity is lost by the use of other oils that also contain antioxidant compounds (e.g. olive oil) for the control group, and future studies would benefit from additional trial arms with alternative placebos. Animal studies have also readily employed antioxidant compounds to prevent or reverse the effects of specific pollutants. These studies have been valuable for investigating specific endpoints that cannot be easily addressed in human studies. In most cases, antioxidants were extremely effective in preventing the effects of air pollution exposure. Even with publication bias, the efficacy of antioxidants in preventing the cardiovascular effects of pollutant exposure in both animals and human studies confirms the key role of oxidative stress in these actions. Other agents that have been explored include a range of natural compounds in with anti-inflammatory properties, beta-blockers and statins. Some of these agents (e.g. beta blockers) are likely to have clear actions on specific cardiovascular parameters (e.g. cardiac), whereas others (e.g. statins) may have actions on different facets of the cardiovascular system (e.g. circulating vascular adhesion molecules). Beneficial effects of other medications that could ameliorate some of the cardiovascular effects of air pollution may have been overlooked as cardiovascular parameters were not the principal focus of the research study. For example, corticosteroids and leukotriene inhibitors have been shown to prevent some the pulmonary effects of PM exposure (Delfino et al., 2006). It has not been established if inhibition of pulmonary inflammation could also lead to an amelioration of some cardiovascular parameters that are mediated by transmission of pollution-induced inflammation into the systemic circulation (see Fig. 2). In conclusion, further studies are now needed to confirm the findings of the modest numbers of studies showing beneficial actions of pharmacological agents and, ideally, extend the range of cardiovascular parameters investigated. If possible, further efforts should be made to ascertain if the compounds are specifically targeting the direct actions of pollutants or having a more general cardiovascular benefit independent of pollutant exposure.

While the aim of this review is to consider pharmacological methods as a means to explore biological mechanisms, these studies inevitably ask the question as to whether medication should be used as a means to protect individual against the actions of air pollution. Medicines should not replace efforts to remove the sources of air pollution. However, implementation of air pollution reduction measures, policy changes and lifestyle changes inevitably require time. Subsequently, there could be a place for medicinal interventions that can lessen the biological effects of air pollution in the intervening period. This is especially the case for those that may be at greater risk (e.g. the young, the elderly, those with pre-existing cardiorespiratory disease, pregnant mothers) and/or those who have an unavoidable high exposure to pollutants. Reduction of risk factors and healthy living will benefit those living in clean air as well as polluted air. Nonetheless, adopting an antioxidant-rich diet may be especially beneficial for those who are particularly risk of the effects of pollution. Additionally, antioxidant supplements are readily-available, low cost and largely innocuous (at moderate doses at least), and the evidence described in this review suggest that is certainly some potential for these supplements to lessen the cardiovascular effects of air pollution. The use of other types of medication to protect against air pollution will require further research. For those who have diagnosed cardiovascular conditions, medication such as beta-blockers or statins may indeed have additional benefits in those exposed to high air pollution. However, at present, there is insufficient evidence (and indeed it would be negligent) to recommend the use of these medicines to counter the effects of air pollution in those with mild conditions that would otherwise not be treated. Additionally, while several medicines appear to have beneficial effects, the evidence is not strong enough for medicines to be viewed as being ‘protective’, especially against cardiovascular events. Interventions, such as facemasks, vehicle cabin filters or indoor air purifiers, may have a role here given emerging evidence suggesting that these can be effective at ameliorating the cardiovascular effects of air pollution to some degree (Chen et al., 2015; Guan et al., 2018; Langrish et al., 2012; Langrish et al., 2009; Liu et al., 2018; Pettit et al., 2015; Yang et al., 2018), although further research is needed to establish the level of ‘protection’ they provide. This nuance of ‘protection’ versus ‘benefit’ requires careful messaging to prevent a false-sense-of-security that could lead to behavioural changes where an individual inadvertently places themselves at greater risk (e.g. greater exposure to air pollution). Additionally, if new research does indeed suggest that certain medicines could be used as therapeutic interventions in susceptible individuals, careful consideration would be needed to address which facets of cardiovascular health are targeted (e.g. decelerating the disease process, or minimising risk of a cardiovascular event) and the strategy by which the therapy would need to be taken (e.g. patient criteria, long-term prophylactic use versus pre-emptive ad-hoc use before a particular activity/exposure).

Overall, this review highlights that there is now a growing body of evidence demonstrating that antioxidants, and potentially other therapies, can ameliorate the actions of air pollution exposure throughout the cardiovascular system. A complex network of interacting mechanisms accounts for the multiple actions of particulate air pollution on the cardiovascular system. Pharmacological studies have been a valuable complementary tool in dissecting these mechanisms. An improved understanding of these mechanisms will offer insight for a number of pertinent questions such as which pollutants are predominantly responsible for driving health effects, and who is especially susceptible? Pharmacological tools will be a vital addition to the scientific arsenal to address these matters and build on the strong foundations of scientific understanding that will ultimately support strategies to reduce the substantial burden on health from air pollution.

## Disclosures

None. The author declares that he has no competing interests.
